# Impacts of urban carbon dioxide emissions on sea-air flux and ocean acidification in nearshore waters

**DOI:** 10.1371/journal.pone.0214403

**Published:** 2019-03-27

**Authors:** Devon Northcott, Jeff Sevadjian, Diego A. Sancho-Gallegos, Chris Wahl, Jules Friederich, Francisco P. Chavez

**Affiliations:** 1 Monterey Bay Aquarium Research Institute, Moss Landing, CA, United States of America; 2 Scripps Institution of Oceanography, La Jolla, CA, United States of America; 3 Stanford University, Stanford, CA, United States of America; University of Bologna, ITALY

## Abstract

Greatly enhanced atmospheric carbon dioxide (CO_2_) levels relative to well-mixed marine air are observed during periods of offshore winds at coastal sensor platforms in Monterey Bay, California, USA. The highest concentrations originate from urban and agricultural areas, are driven by diurnal winds, and peak in the early morning. These enhanced atmospheric levels can be detected across a ~100km wide nearshore area and represent a significant addition to total oceanic CO_2_ uptake. A global estimate puts the added sea-air flux of CO_2_ from these greatly enhanced atmospheric CO_2_ levels at 25 million tonnes, roughly 1% of the ocean’s annual CO_2_ uptake. The increased uptake over the 100 km coastal swath is of order 20%, indicating a potentially large impact on ocean acidification in productive coastal waters.

## Introduction

The increase in atmospheric carbon dioxide (CO_2_) from the burning of fossil fuels has been well documented by decades of measurements from the top of Mauna Loa on the big island of Hawaii [1]. Keeling chose the iconic Mauna Loa site because it rises into the free troposphere that is less affected by local sources of carbon pollution. Oceanographers often use well-mixed atmospheric values from similar sites or global models to estimate the sea-air exchange of CO_2_ [2]. Global flux estimates find the current ocean uptake to be about two petigrams (two billion tonnes) of carbon per year [2,3]. This steady uptake of atmospheric CO_2_ by the oceans results in the so-called phenomenon of ocean acidification [4]. However, studies of atmospheric CO_2_ concentrations in urban environments have shown considerable enhancements of CO_2_ in city centers, especially in the early morning [5,6,7], an effect known as the urban CO_2_ dome. Agricultural practices can also impact local atmospheric CO_2_ on a diurnal cycle with large nighttime increases due to respiration and daytime decreases associated with photosynthesis [8,9]. Near coastlines these elevated levels of CO_2_ might impact marine air via atmospheric circulation and therefore increase the flux of CO_2_ into nearshore waters enhancing ocean acidification. Here we present novel observations from nearshore moorings and an autonomous sea surface vehicle that show the magnitude of urban CO_2_ pollution and allow us to calculate the contribution to sea-air fluxes from this previously unquantified source.

The diurnal cycles in CO_2_ concentration over urban environments has been shown to peak just before sunrise at 4-5am and reach a minimum at around 4pm in the Los Angeles basin [7]. Modeling has shown similar CO_2_ dome effects over the San Francisco and Monterey Bay areas [10]. However, little attention has been paid to the advection of these urban CO_2_ domes over oceans and resulting impact on sea-air CO_2_ flux. Monterey Bay is ideally situated for such observations, as large urban and agricultural areas in the Salinas and Silicon valleys are nearby, and a strong diurnally varying component in winds [11,12] can transport high levels of locally produced atmospheric CO_2_ over the ocean. In the Monterey Bay Area the urban and agricultural CO_2_ dome should reach its maximum concentrations at roughly the same time as the peak of the offshore phase of the diurnal wind cycle, leading to the advection of high CO_2_ air from land sources over the coastal ocean.

As indicated above, traditional estimates of sea-air CO_2_ flux are not able to quantify this nearshore phenomenon because they have a temporal or spatial scale that is too coarse to resolve these diurnally varying CO_2_ anomalies. Therefore, the impact of a potentially significant source of atmospheric CO_2_ on fluxes into the ocean has not previously been estimated. These impacts are magnified near urban or agricultural areas with strong offshore winds that can advect heavily polluted air over marine waters. Here we use high temporal resolution (1 hour over years) timeseries from multiple autonomous ocean based sensor platforms (moorings and surface vehicles) to provide a detailed assessment of the impacts of these high frequency variations in atmospheric CO_2_ concentration on sea-air CO_2_ fluxes in the nearshore environment.

## Material and methods

### Data sources

**Fig 1:**
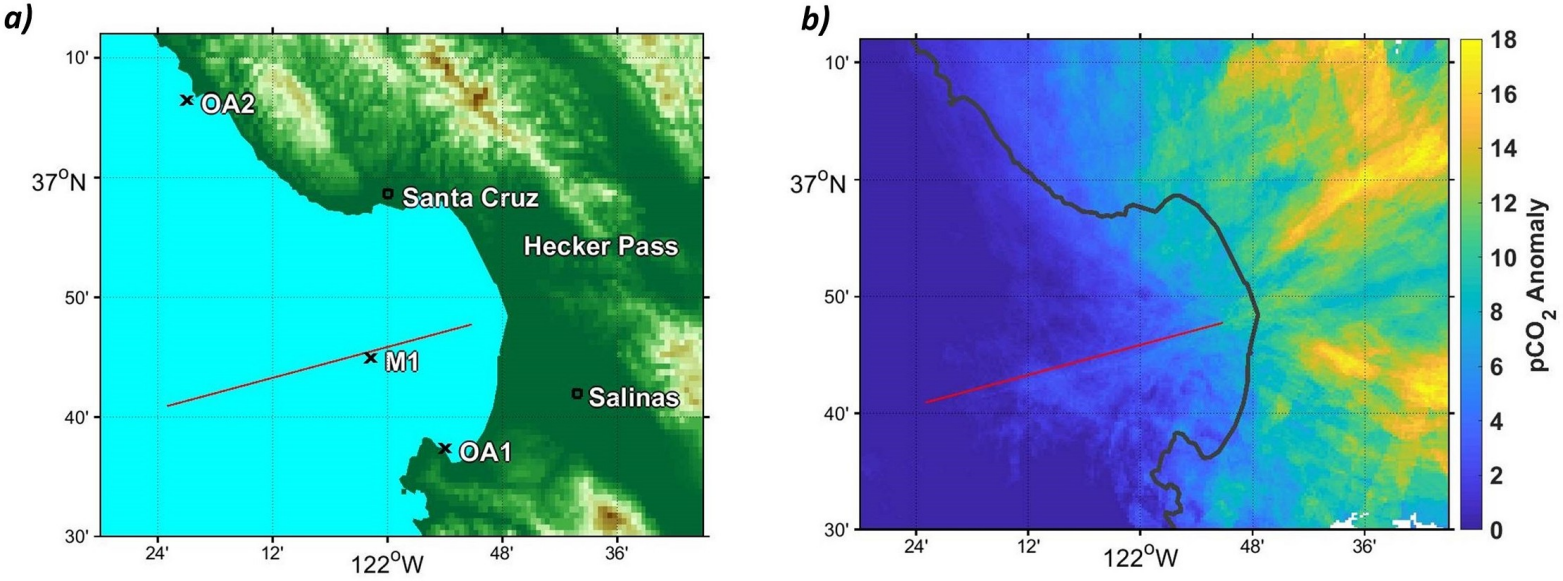
Location of data sources and predicted terrestrial sources of atmospheric CO_2_ from wave glider data. a) Topography of the Monterey Bay area showing the location of platforms used in this study, as well as the Hecker pass and Salinas Valley, two major terrestrial sources of enhanced atmospheric CO_2_ to Monterey Bay waters. The Monterey Bay timeseries (MBTS) line is also shown. b) Source regions for atmospheric CO_2_ anomalies (the difference between well-mixed marine air and enhanced atmospheric CO_2_; see “Anomaly Calculations” section in Methods) traced back from wave glider measurements along the MBTS Line (Fig 1A). Anomalies are particularly high in the Salinas Valley and Hecker pass areas. Anomalies are calculated for wave glider observations within 5km of the MBTS Line, and then propagated backwards for 6 hours along a path defined by wind direction and speed at the wave glider. A 5 degree uncertainty cone is drawn around the path, and all paths are averaged to yield the final figure. Coastline data republished from the Global Self-consistent, Hierarchical, High-resolution Geography Database (GSHHG) under a CC BY license, with permission from Dr. Paul Wessel, original copyright 1996.

Data was collected from four different platforms operated by the Monterey Bay Aquarium Research Institute (MBARI) in the Monterey Bay region. Of these, three were moorings (OA1, OA2 and M1; Fig 1A) and one was an autonomous surface vehicle (Liquid Robotics Wave Glider [13]). All except M1 were outfitted with Airmar WX200 ultrasonic wind sensors which record wind speed and direction, Licor non-dispersive infrared gas analyzer CO_2_ instruments which were developed to measure CO_2_ concentrations in the air and water [13,14], and Seabird temperature and salinity sensors (herein CTD). M1 used an Aandara sonic anemometer together with the same CO_2_ instrument and CTD. Measurements were averaged hourly in the final analysis. Wave glider measurements were taken within 5km of the Monterey Bay Time Series (MBTS) Line, a transect extending from Moss Landing out 50km along the Monterey canyon (Fig 1, red line). The moorings are located 1.5km offshore of Año Nuevo north of Santa Cruz, California (OA2), 300m off shore off of Monterey, California (OA1), and 20km offshore of Moss Landing, CA, in the center of the Monterey Bay (M1). The OA moorings are at 20 m and M1 at 1000 m depth. Wave glider data from station M, a station 220km offshore of San Luis Obispo, California (123 W 35.14 N) were also considered. Data were taken between 2013 and 2018, and all records were used, except in cases of dropout of CO_2_ sensors, wind measurements, or CTD sensors. M1 recorded 1417 days of data between September 2013 and April 2018, OA1 recorded 853 days between January 2014 and July 2017, OA2 sampled 461 days between May 2015 and July 2017, and the wave glider recorded 182 days of measurements on the MBTS Line between March 2014 and March 2018.

### Winds

**Fig 2:**
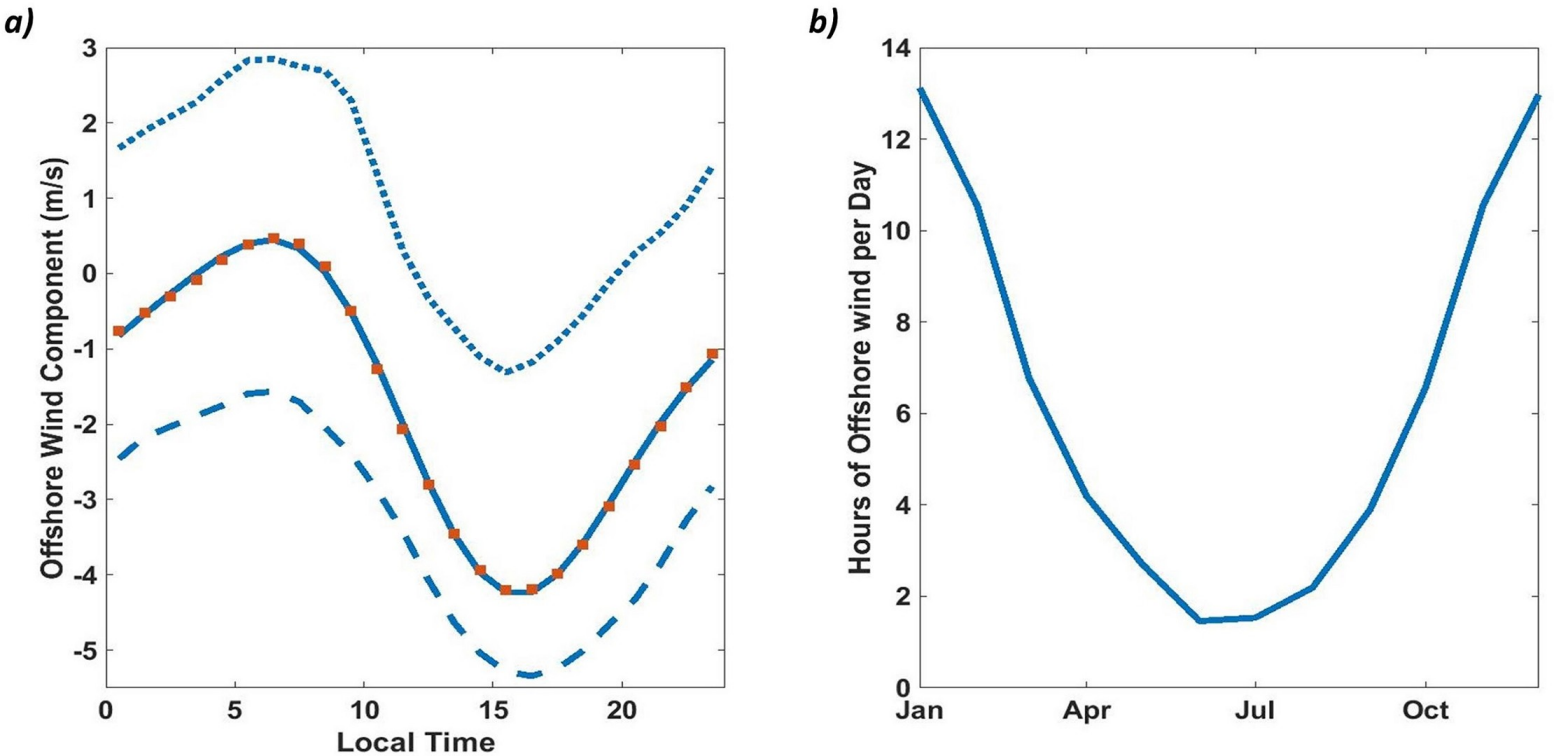
Diurnal and annual cycles of offshore winds at the M1 mooring. a) Offshore (easterly) component of winds at the M1 mooring (see Fig 1A for location) averaged hourly. Positive (negative) values represent offshore (onshore) winds. The dotted line shows January data, representing the seasonal peak in offshore wind duration and amplitude. The dashed line represents July winds, while red squares represent full year averages, and the solid line shows the best fit to this data. Maximum offshore winds are observed at 6-7am local time. Phasing of the fit remains consistent year-round. b) Duration of offshore (0–165 degrees) winds (hours) by month over the 29 year record from the M1 mooring. There is strong correlation between daily duration of offshore wind and sea-air fluxes driven by atmospheric CO_2_ anomalies (see text).

An analysis of the phasing and length of offshore wind events was performed using the full 29 year M1 wind record. The east-west component of the winds was isolated, and binned by hour (Fig 2A). This analysis was run on both the full record, and by month to examine seasonal variations in the phasing and strength of land-sea breezes. While the strength of offshore winds varied by month the phasing of the cycle did not. The number of hours of offshore wind (wind direction between 0 and 165 degrees) on each fully sampled day in the record was then calculated, and these results were binned by month (Fig 2B) to give a measure of the seasonality of the persistence of offshore wind.

### Atmospheric CO_2_ anomaly calculations

In this paper “atmospheric CO_2_ anomaly” refers to a deviation of atmospheric CO_2_ values from a baseline CO_2_ concentration that reflects a well-mixed atmosphere. To calculate these baseline values, a rolling median filter was applied to CO_2_ measurements with duration of 15 days for moorings and 4 days for the wave glider. The filter window for the wave glider data was used because the short mission durations and high mobility of the platform lead to poor estimates of baseline pCO_2_ values when the full 15 day filter window was applied. CO_2_ measurements taken when the wind was blowing from the open ocean for more than 3.5 hours were used to calculate baseline values to ensure that baselines were representative of well-mixed marine air CO_2_ concentrations. This baseline was then subtracted from the timeseries of atmospheric CO_2_ values to yield a timeseries of atmospheric CO_2_ anomalies. Comparison of the baseline pCO_2_ values with NOAA’s Global Greenhouse Gas (GHG) Reference Network [15] Trinidad Head station (r^2^ = 0.71), as well as CarbonTracker [16] modeled values (r^2^ = 0.56), showed good agreement. We used the calculated baseline pCO_2_ values since these sources only run until December 2016 while our timeseries extends through early 2018.

### CO_2_ Fluxes

The net sea-air CO_2_ flux was estimated using the equation:

$$FCO_{2}=k*S*\Delta pCO_{2}$$ (1)

where k is the gas transfer velocity[17], S is the solubility of CO_2_ in seawater [18] and Δ*pCO*_2_ is the difference between pCO_2_ water and pCO_2_ air. Gas transfer velocity is parameterized as a function of wind speed squared, while solubility is a function of water temperature and salinity. The common convention is used whereby a negative flux indicates CO_2_ transfer into the ocean (a sink); while a positive flux indicates release of CO_2_ into the atmosphere (a source).

### Wave glider atmospheric CO_2_ anomaly predictions

In order to construct a high resolution map of enhanced atmospheric CO_2_ sources, anomalies were calculated for each wave glider data point. At ten minute intervals the wind speed and direction were then used to calculate the source position of the wind; this process was repeated additively over the six hours prior to any given air CO_2_ measurement to construct a probable path for the measured air parcel. This method ignores local variation in wind direction and speed, mixing, and in particular excludes the effects of topography on offshore winds. In order to reduce these effects, all pixels within a five degree cone around the calculated path were assigned the value of the calculated anomaly, and added to the developing composite image containing all previous tracks. When complete the image was then divided by the number of tracks intersecting each pixel, producing an average map of probable atmospheric CO_2_ anomaly sources and their strengths. This method should not be seen as providing exact locations or intensities of anomaly sources but it is straightforward and provides a general picture of the origin of strong atmospheric CO_2_ anomalies.

### Fluxes driven by atmospheric CO_2_ anomalies

To estimate the contributions of the atmospheric CO_2_ anomalies to sea-air fluxes, two flux calculations were made. The first used the well-mixed oceanic air baseline CO_2_ concentrations calculated as described above. This dataset represents atmospheric CO_2_ concentrations used in studies of sea-air flux that rely on modeled or well-mixed atmospheric CO_2_ values. This timeseries of fluxes was subtracted from a second timeseries calculated using the observed pCO_2_ air concentrations. The difference between these two timeseries gives a measurement of flux due to our calculated atmospheric anomalies in air pCO_2_. This method preserves the convention of negative values indicating increased transport of CO_2_ into the ocean.

## Results

**Fig 3:**
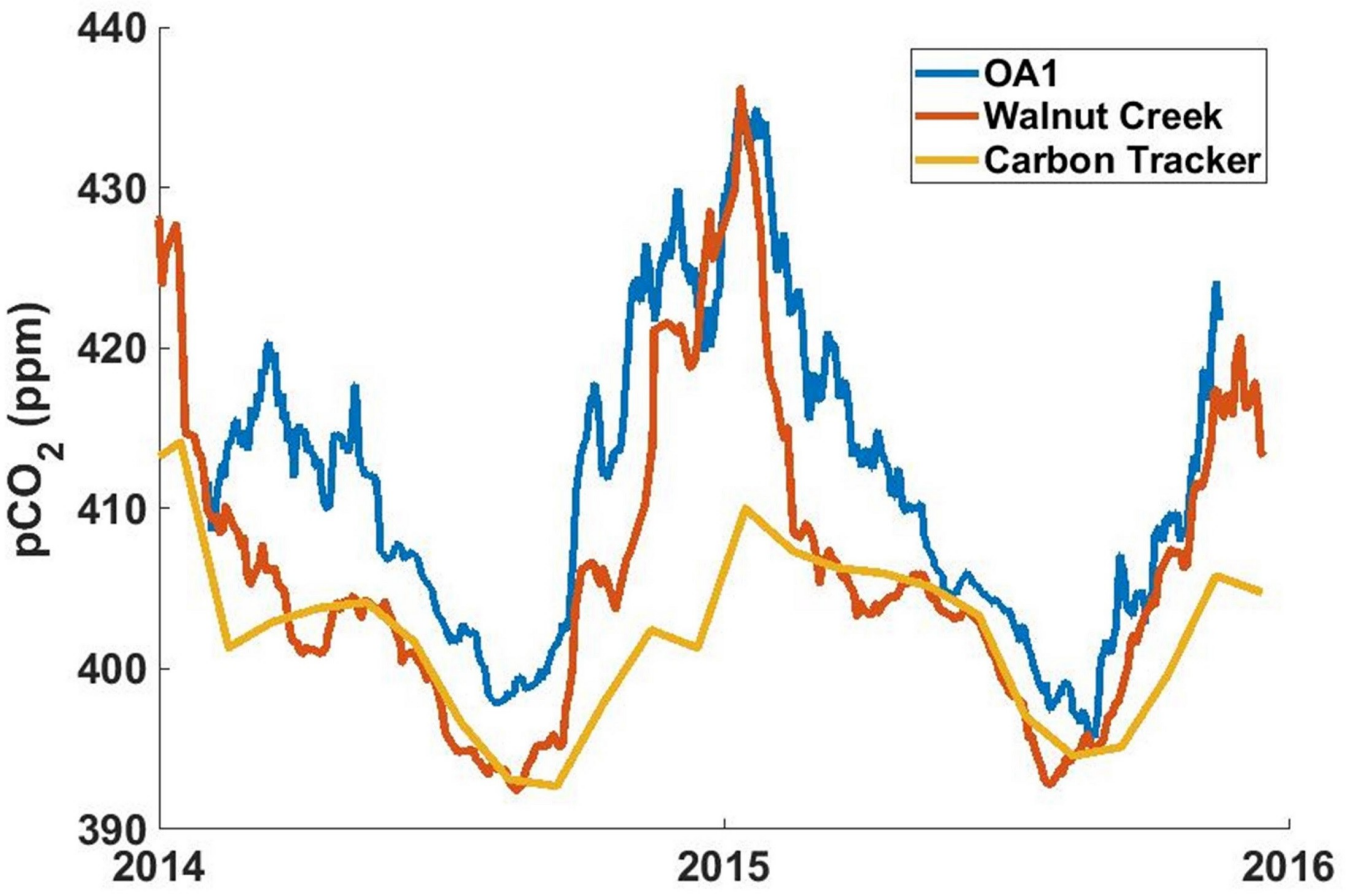
OA1 atmospheric pCO2 vs nearby terrestrial measurements and modeled values. OA1 mooring atmospheric CO_2_ plotted with atmospheric CO_2_ data measured at the NOAA GMD tower network Walnut Creek station (WGC) (Andrews, Kofler, Bakwin, Zhao, & Trans, 2009) and monthly modeled CarbonTracker CT2016 CO_2_ at OA1 (Peters, et al., 2007). Tick marks represent the beginning of each year. OA1 and Walnut Creek data include periods of large atmospheric CO_2_ anomalies, and were smoothed with a 15 day moving average. The CO_2_ concentration at OA1 tracks that from Walnut Creek, a nearby urban location, and both differ significantly from the model that represents well-mixed marine atmospheric CO_2_ concentration. Atmospheric CO_2_ data collected during long periods of onshore winds (not plotted) correlates well to the CarbonTracker modeled values (r^2^ = 0.56).

Significant positive anomalies in atmospheric CO_2_ are detected on all platforms during periods of offshore winds. A time series of atmospheric CO_2_ from the OA1 mooring over 2014 and 2015 illustrates the extent of these anomalies (Fig 3). In Fig 3 a timeseries of atmospheric CO_2_ from a nearby urban terrestrial station (Walnut Creek) [16] and clean marine air from Carbon Tracker [15] are plotted together with OA1. The similarity between OA1 and Walnut Creek is striking as are the mostly winter time increases in atmospheric CO_2_ at both these sites relative to well-mixed marine air.

**Fig 4:**
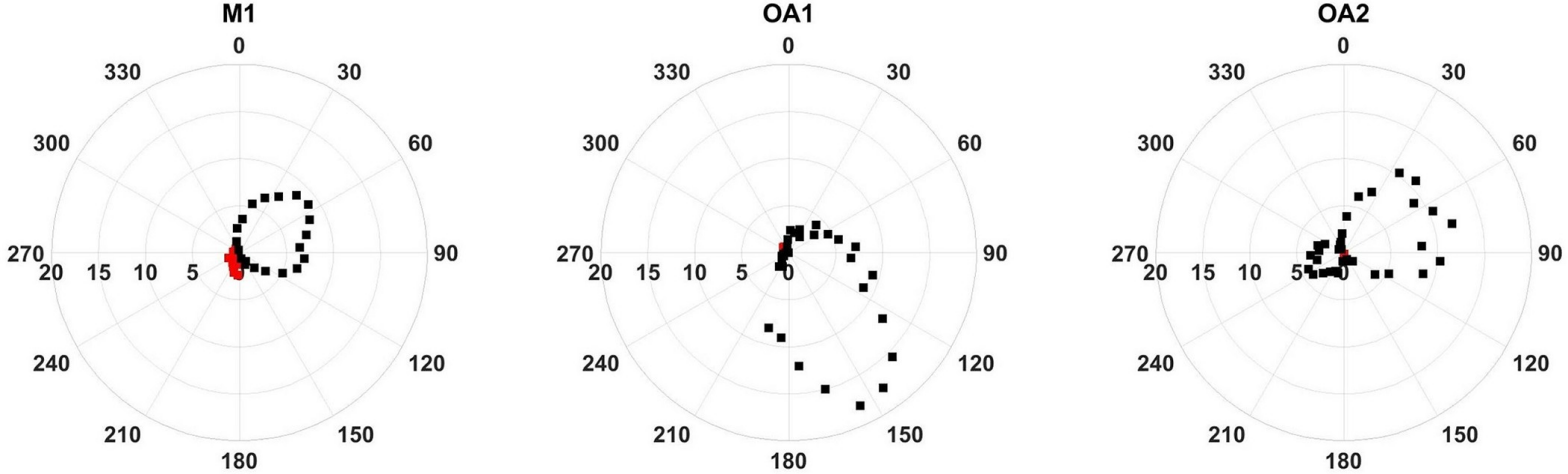
CO_2_ atmospheric anomalies (ppm) at the moorings averaged by wind direction. Anomalies are calculated as deviations from a 30-day median filtered timeseries. Positive anomalies are in black, while negative anomalies are in red. Maximum positive atmospheric CO_2_ anomalies are found at all moorings when winds originate over land. M1 shows a double peak in anomalies related to wind direction from Hecker Pass and Salinas Valley. OA1 is very nearshore, and displays the strongest anomalies from air originating from the city of Monterey directly to its south, with a secondary peak pointing eastward toward the Salinas valley. OA2 winds are prevailing from the ocean and display weak topographic amplification, resulting in smaller anomalies that originate from urban locations in Silicon Valley.

Using a basic advection model in conjunction with wave glider data (see methods) we found that the largest atmospheric CO_2_ anomalies detected along the MBTS line originate from the Salinas valley and Hecker Pass (Fig 1B). These topographic features connect marine waters in the Monterey Bay region with large urban or agricultural areas inland and suggest that topography steers air with enhanced levels of CO_2_ toward Monterey Bay. Hecker Pass represents a break in the mountains through which pollution from Silicon Valley can reach the coast, while the Salinas Valley contains urban centers and large agricultural fields. Binning atmospheric CO_2_ anomalies measured at moorings by wind direction confirms sources at those locations. (Fig 4). The M1 mooring, which is situated at the center of the MBTS Line, shows a double peak in atmospheric CO_2_ anomalies corresponding Hecker Pass (NE, 60 degrees) and the Salinas Valley (E, 100 degrees). OA1 is also impacted by CO_2_ anomalies during periods of easterly winds from these topographic features. However, its largest anomalies are registered when winds blow from ~150 degrees. OA1 is situated only a few hundred meters north of the city of Monterey and the monthly averages at this buoy were much better correlated with the urban CO_2_ measurements from Walnut Creek (r^2^ = 0.78), than with CarbonTracker modeled atmospheric CO_2_ (r^2^ = 0.47) (Fig 3). This suggests that atmospheric CO_2_ at OA1 is strongly influenced by air emanating from the city of Monterey. Meanwhile the largest atmospheric CO_2_ anomalies at OA2 off Año Nuevo came from the northeast, directly from the Silicon Valley area (Fig 4).

**Fig 5:**
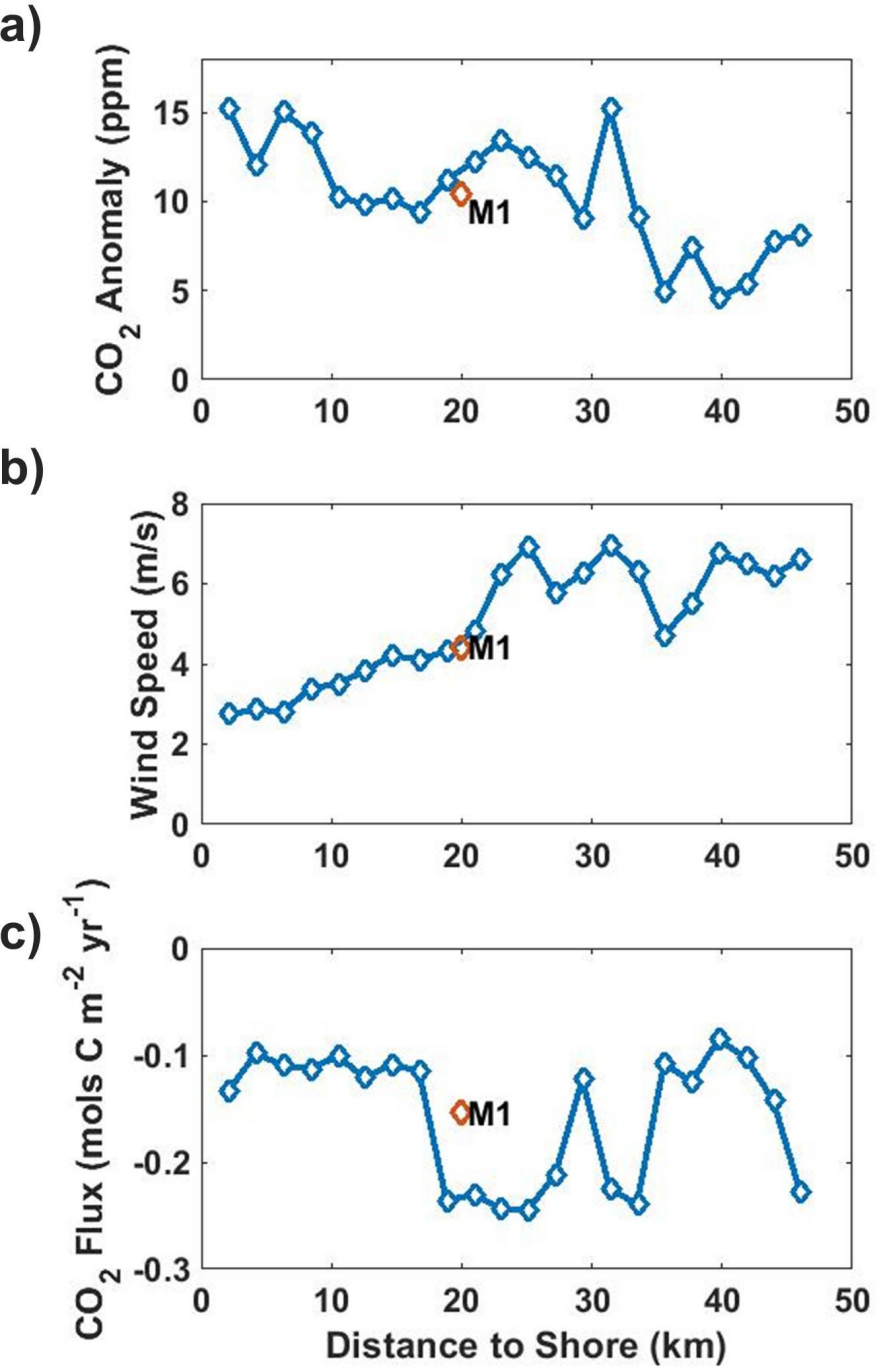
CO_2_ anomalies, wind speed and sea-air flux anomalies along MBTS line and M1. MBTS Line wave glider and M1 measurements of (a) atmospheric CO_2_ anomalies, (b) average wind speed, and (c) anomaly driven sea-air CO_2_ flux, calculated during offshore wind events, and plotted against distance from shore. Anomalies are calculated by subtracting the median of persistent onshore winds. Sampling effort is biased toward the shoreward bins. A large increase in air-sea fluxes (negative flux values) is observed between 20 and 35 km offshore, driven by an increase in wind speeds with distance offshore. Meanwhile, CO_2_ anomalies drop off with distance offshore. There is good agreement between the wave glider and M1 data even though these were collected over different time periods and at different resolutions.

Average anomalies at OA1 were close to double those at M1 during periods of offshore winds. This indicates a reduction in the strength of atmospheric CO_2_ anomalies with distance from shore. The wave glider provides the perfect platform to further explore this relationship. Frequent wave glider measurements extend 50km out to the end of the MBTS Line, and average anomalies of 6-10ppm are detected at the end of this line during periods of offshore winds. This shows that during offshore wind events the plume of high CO_2_ air extends at least 50km from shore, although its CO_2_ content is reduced on average from the 15 ppm anomalies seen nearshore (Fig 5). An outer limit is seen on anomaly propagation when data from station M is considered. This station is 220km offshore and routinely occupied by the wave glider. No significant atmospheric CO_2_ anomalies associated with offshore wind events are detected at this location, with all reported anomalies being within the uncertainty of the sensor. Since enhanced CO_2_ levels are transported offshore by diurnal land-sea breezes, the area in which these high CO_2_ levels can be observed depends on the areal extent of the land-sea breezes. The strength of offshore winds in central California varies diurnally [11,12], and the average duration of an offshore wind event at M1 is relatively short at ~6 hours. Average offshore wind speeds were 4.4 m/s, so assuming CO_2_ is transported offshore at that wind speed for the duration of an offshore wind event, anomalies should rarely be detected more than 100km from the coast. This agrees with more thorough modeling and remote sensing studies that have concluded that the seaward influence of diurnal land breezes extends roughly 100km offshore along much of the west coast of the United States [19,20].

**Fig 6:**
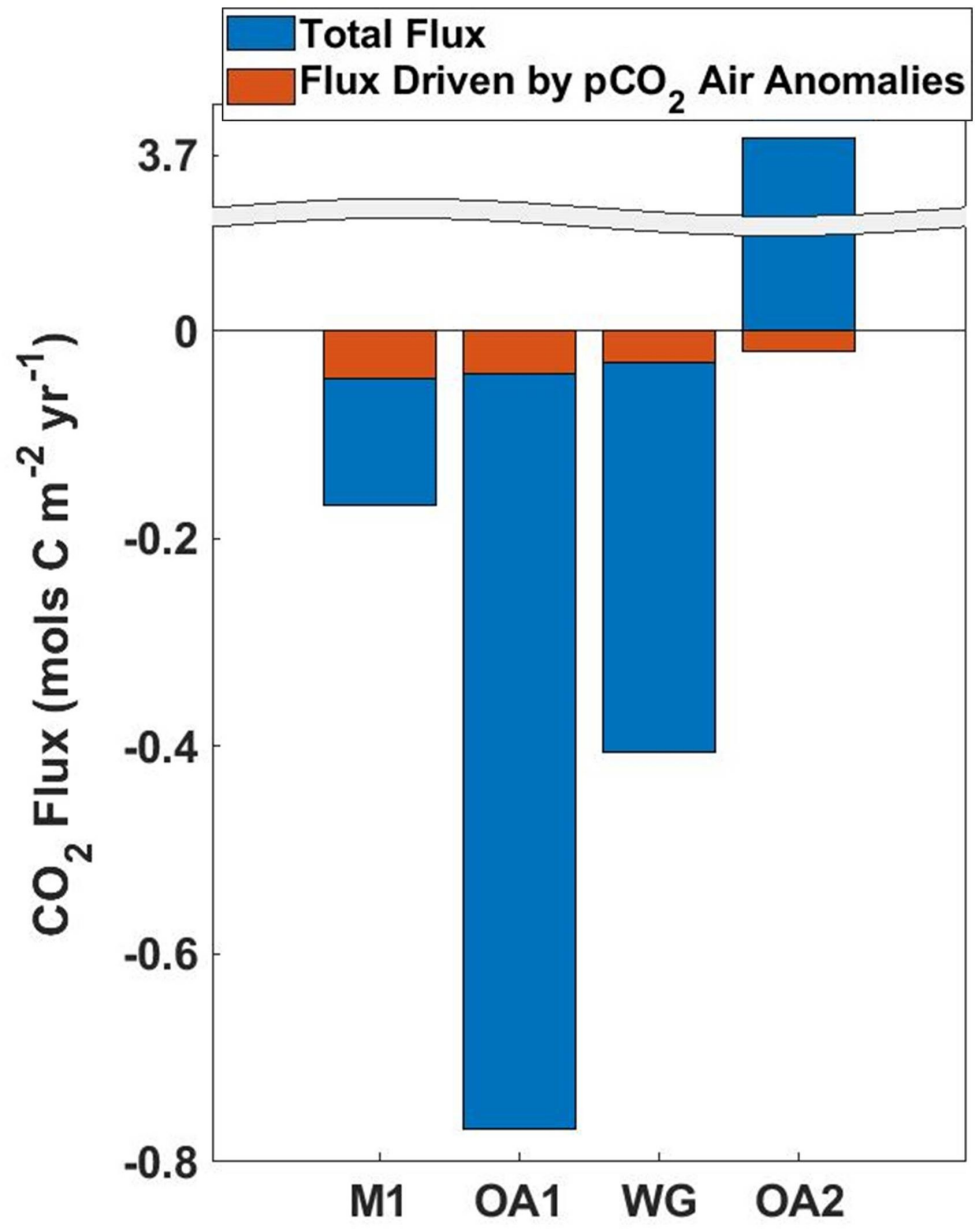
Yearly flux at all platforms. Sea-air CO_2_ flux calculated using well mixed marine air (blue portion) together with that driven by enhanced atmospheric CO_2_ (red portion) across all platforms. The striking spatial variability is evident, as is the relatively constant atmospheric CO_2_ anomaly driven flux. Negative (positive) values represent uptake (loss) of CO_2_ by the ocean. Increased transfer of CO_2_ into the ocean due to anomalously high atmospheric CO_2_ was estimated for all platforms.

Total sea-air fluxes varied widely across the study area, largely owing to upwelling processes. OA2 sits directly in the upwelling plume off Año Nuevo where water high in carbon dioxide is brought to the surface during upwelling [13,21,22]. As a result, CO_2_ flux is large and positive in recently upwelled water indicating that freshly upwelled waters are a considerable source of atmospheric CO_2_. M1 is downstream from the upwelling plume, in an area where phytoplankton have increased and via photosynthesis converted much of the upwelled carbon dioxide into organic carbon. As a result average fluxes are slightly negative in this area, indicating that on an annual basis M1 is a weak sink for carbon. Inside Monterey Bay and along the southern edge fluxes are increasingly negative as the slower circulation allows phytoplankton to bloom and further reduce pCO_2_. OA1 and the MBTS Line average, which is weighted to the inshore environment owing to uneven sampling effort, reflect this greater sink of atmospheric CO_2_ (Fig 6).

Sea-air CO_2_ fluxes due to enhanced atmospheric CO_2_ were similar across all platforms. The highest atmospheric CO_2_ anomaly driven fluxes were recorded at M1, reaching 0.046 mols C m^-2^yr^-1^. While atmospheric CO_2_ anomalies were higher at OA1 owing to this mooring’s close proximity to land and urban areas, a lower average offshore wind speed and a lower incidence of offshore winds reduced the effects of the higher atmospheric CO_2_ on fluxes, resulting in an anomaly driven flux of 0.041 mols C m^-2^yr^-1^. The total annual flux along the MBTS Line was 0.030 mols C m^-2^yr^-1^. (Fig 6) Average fluxes were reduced over the whole line relative to M1 owing to very low wind speeds close to shore and a drop-off in anomalies beyond 35km from shore (Fig 5). OA2 recorded the lowest average anomalies, as well as the lowest percentage of offshore wind hours, but still displayed wind driven CO_2_ flux anomalies of 0.019 mols C m^-2^yr^-1^ (Fig 6). The averaged offshore profile of anomaly driven sea-air fluxes was dependent on both wind speed and atmospheric CO_2_ anomalies, and was depressed inshore where wind speeds are low, and offshore where wind speed are high but atmospheric CO_2_ anomalies are lower. The combination of these factors leads to a large increase in sea-air fluxes between 20 and 30 km offshore, where relatively stronger offshore winds combine with high atmospheric CO_2_ anomalies (Fig 5).

**Fig 7:**
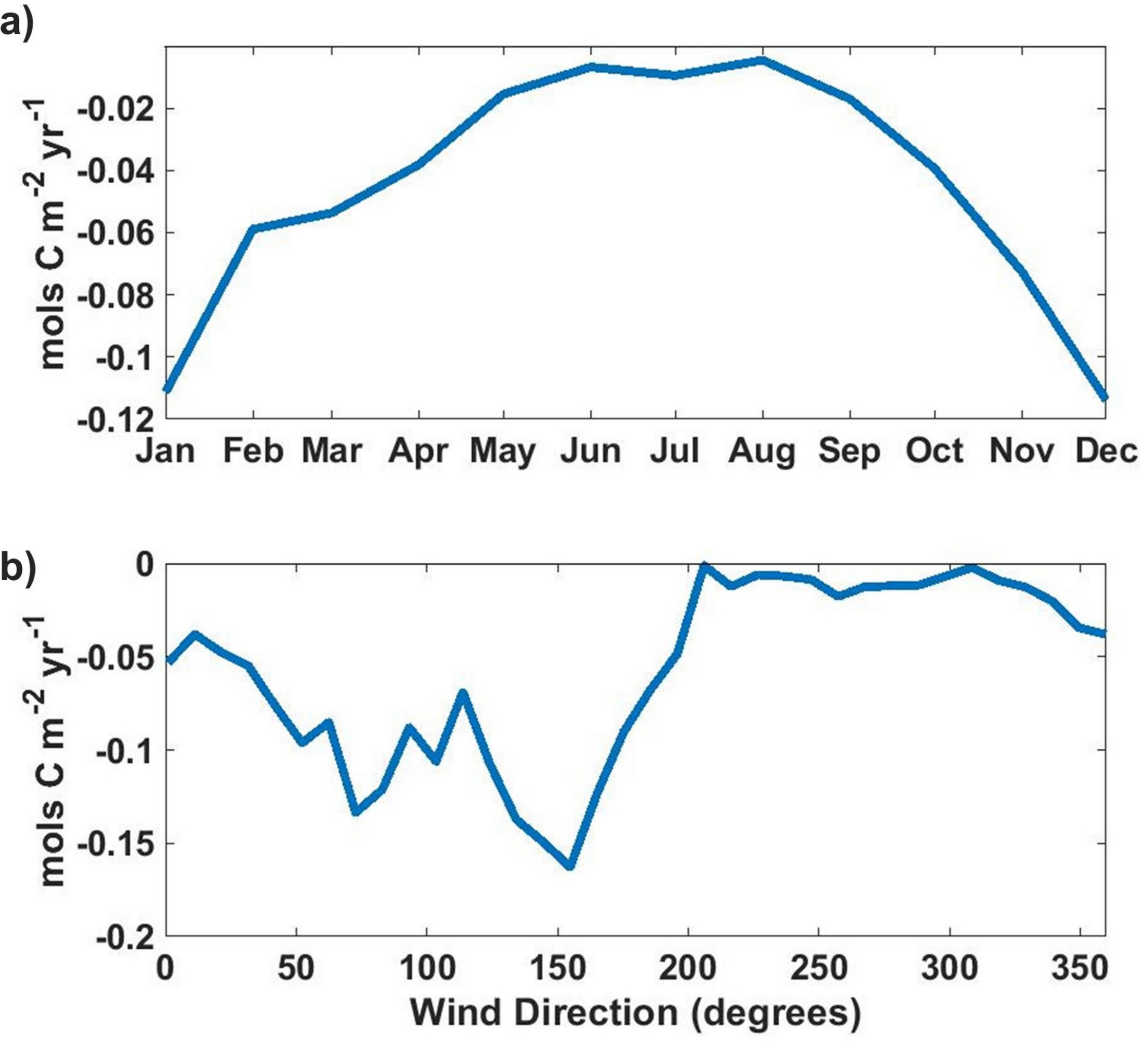
OA1 anomaly driven flux by month and wind direction. Atmospheric CO_2_ anomaly driven sea-air flux averaged by month (a) and by wind direction (b) at OA1. Negative values indicate CO_2_ flux into the ocean. Atmospheric CO_2_ anomaly driven fluxes are strongest in the winter months when offshore winds are stronger and more frequent. Anomaly driven fluxes are restricted to periods of offshore winds.

Sea-air CO_2_ fluxes due to atmospheric CO_2_ anomalies undergo a strong seasonal cycle with increased offshore wind duration (Fig 2B) in the winter months driving larger and more frequent anomalies at all platforms. OA1 exhibits a particularly strong seasonal cycle, as strong wintertime storm winds are oriented to bring polluted air directly from the city of Monterey onto the buoy (Fig 7). At M1, where southerly storm winds come off the open ocean, a strong seasonal cycle is still present due to higher incidence of easterly and southeasterly offshores during the winter season. (Fig 2B)

## Discussion

Studies of the role of the oceans in the global CO_2_ budget often use low-resolution information on atmospheric CO_2_ concentration to estimate sea-air CO_2_ fluxes. Large scale models or relatively few stations representative of well-mixed marine air concentrations are consistently used in flux calculations [2,23]. Observations from autonomous platforms in coastal California show that these clean air open ocean carbon dioxide concentrations are often not representative of carbon dioxide concentrations in the air nearshore. Nearshore areas experience significant enhancement from land sources and this impact can extend on the order of 100km from the coast. These atmospheric CO_2_ anomalies, relative to well-mixed atmospheric air, contribute to sea-air fluxes regardless of the underlying oceanographic and atmospheric conditions. The association of diurnal winds to these atmospheric anomalies clearly ties them to terrestrial sources. The high frequency variability also makes it difficult to model these terrestrial sources from sparse temporal or spatial resolution datasets. High-resolution measurements from autonomous platforms allow us to make an initial estimate of the impacts of elevated atmospheric CO2 levels from highly polluted terrestrial sources on sea-air fluxes.

The combination of offshore winds and terrestrial carbon sources drive large gradients in sea-air CO_2_. However, the relatively weak offshore winds in the Monterey Bay greatly constrain CO_2_ flux since it is parameterized to approach zero under very low wind speeds. This is a reasonable approximation in the open ocean where average wind speeds are rarely very low; the current parameterization may not apply when wind speeds are less than 3m/s, seriously underestimating sea-air flux at low wind speeds [17]. Average offshore wind speeds recorded along the Monterey Bay time series line never exceed 7m/s, and nearshore where CO_2_ anomalies are largest wind speeds were around 3m/s. While open ocean fluxes may be accurately predicted using current sea-air flux models, our observations highlight the difficulty of using these same models in the nearshore realm, where wind speeds are lower.

The atmospheric CO_2_ anomalies described above should occur anywhere that urban or agricultural areas are found near the coast, and the winds are such that they carry terrestrial sources over the marine environment. The diurnal land-sea breezes that contribute to offshore CO_2_ transport observed in Monterey Bay are a widespread phenomenon worldwide [20]. While the contributions of direct CO_2_ pollution to sea-air fluxes is modest, areas with stronger or more frequent offshore wind events, or larger urban centers situated closer to the water’s edge should experience more significant fluxes. If the wave glider measurements in the Monterey Bay area are taken as a reasonable estimate of average anomaly driven CO_2_ fluxes within 100km of land we can estimate the impact of increased atmospheric CO_2_ globally. We calculated that coastal waters occupy 2.6*10^7^ km^2^ or about 7% of the global ocean so the added contribution to CO_2_ fluxes could be order of 25 million tonnes, or roughly 1% of the ocean’s total estimated CO_2_ uptake[2]. However, since this uptake is concentrated over the 100 km coastal swath the estimated sea-air flux into the ocean in these regions should be increased by around 20%. Clearly, this additional uptake of anthropogenic CO_2_ will have consequences for ocean acidification [4] in nearshore regions where marine biota is concentrated. These same processes should also drive enhanced transport of other terrestrially emitted gases, aerosols, and particles over nearshore marine waters. It is therefore likely that other pollutants are also entering nearshore waters at increased rates. How these enhanced urban sources of pollution will change over time and what are their ecological impacts will need to be assessed by future studies.

## Supporting information

S1 Dataset pCO_2_ Dataset. Dataset containing pCO_2_ air and water measurements, as well as wind speed and direction, sea surface temperature, and salinity at all platforms.
(MAT)
